# Arsenical Poisoning—The “Kloss Case”—Unusual Post-mortem Appearances

**Published:** 1882-10

**Authors:** A. R. Davidson


### Arsenical Poisoning—The "Kloss Case"—Unusual Post-mortem Appearances.

By A. R. Davidson, M. D.

It is well known that the striking changes produced by arsenic are generally confined to the stomach and bowels. Taylor, Woodman, Tidy, and other authorities, assert that their intensity is usually in proportion to the largeness of the dose and the length of time the patient lived after taking the poison. My own experience is that it depends far more upon the conditions existing at the time the poison is taken. If, for instance, a large dose is received during or after a full meal, or mingled with a considerable quantity of food, the chances are favorable to the patient, and if death occurs, after many hours, the characteristic appearances are not nearly so well marked as in those cases in which a comparatively small quantity has been taken on an empty stomach, even if death has taken place in a few hours—in one case where death occurred in two hours, the stomach was found highly inflamed. In these cases the intensity of the inflammation is so great, extending as it does to the duodenum and sometimes through the whole length of the intestines and invariably present in the rectum, that a post-mortem examination can not be made without awakening the suspicion of the presence of an irritant poison.

This condition, too, is persistent for a long time after death. Bodies exhumed six, twelve, and in one case nineteen months after death have shown the stomach well preserved and its mucous membrane retaining an inflammatory redness.

But, on the other hand, where the arsenic has been taken with food, it occasionally happens that death may occur from its effects, and neither the stomach nor intestines present an abnormal appearance. Evidence of poisoning upon post-mortem examination would be here entirely wanting. Such cases are, undoubtedly, very rare, but the possibility of their occurrence is not to be overlooked, as they teach the important fact in legal medicine that the non-existence of striking changes in the alimentary canal after death is no proof that the party has not died from the effects of an irritant poison. It is for this reason that I report the following case:

August 24, 1882, several biscuits were placed in my hands by one of the coroners of Erie county for analysis. Upon examination they were found to contain a quantity of arsenic, equal to about sixteen grains, in each biscuit. A portion of a family, mother and three children, had partaken of the biscuit quite freely at their evening meal, and had all been violently sick with the well-known and characteristic symptoms due to the poison. After twelve hours one of the children, a boy of six years, died. Eight days after, the body was exhumed in my presence; the stomach, kidneys and liver removed; the rectum was opened and found to be free from all indications of inflammation.

The stomach contained a very little whitish fluid, and, upon inspection, the mucous membrane was found to be paler than normal and absolutely free from any semblance of inflammatory action. An analysis proved it to contain a mere trace of arsenic.

The liver and kidneys, treated by Fresenius and Von Babo's method, yielded a little more than half a grain of arsenious acid.

The explanation of these exceptional cases, and of the common ones of recovery after large quantities of poison have been taken with food, is doubtless that the food so envelops the poison that it is prevented from attaching itself to the mucous membrane, and when emesis occurs the arsenic may be entirely thrown off with the contents of the stomach. The amount absorbed before its emetic action is evoked is frequently insufficient to produce death. While a part of the poison is rapidly eliminated by the kidneys, another part seems to be temporarily deposited in the liver and thereby withdrawn from the circulation. From the observations of the late Dr. Geohegan, it appears that the deposit of arsenic in the liver rapidly increases up to about fifteen hours after the poison has been taken, when that organ may contain as much as two grains of arsenic; if the person survives, it then very gradually diminishes in quantity, and entirely disappears in from fourteen to seventeen days.

